# Why women do not ask for information on preconception health? A qualitative study

**DOI:** 10.1186/s12884-016-1198-z

**Published:** 2017-01-05

**Authors:** Renata Bortolus, Nadia C. Oprandi, Francesca Rech Morassutti, Luca Marchetto, Francesca Filippini, Eleonora Agricola, Alberto E. Tozzi, Carlo Castellani, Faustina Lalatta, Bruno Rusticali, Pierpaolo Mastroiacovo

**Affiliations:** 1 Office for Research Promotion, Department of the Hospital Management, Verona University Hospital, Verona, Italy; 2 Multifactorial Disease and Complex Phenotype Research Area, Bambino Gesù Children’s Hospital IRCCS, Rome, Italy; 3 Cystic Fibrosis Centre, Verona University Hospital, Verona, Italy; 4 Clinical Genetics Unit, Fondazione IRCCS Cà Granda Ospedale Maggiore Policlinico, Milan, Italy; 5 National Agency for Regional Health Services, Rome, Italy; 6 Alessandra Lisi International Centre on Birth Defects and Prematurity - ICBD, Rome, Italy

**Keywords:** Preconception health and care, Focus group, Risk factors, Adverse reproductive outcomes, Women of childbearing age, Healthcare professionals

## Abstract

**Background:**

Preconception care involves health promotion to reduce risk factors that might affect women and couples of childbearing age. The risk factors of adverse reproductive outcomes include recognized genetic diseases in the family or the individual, previous congenital diseases, miscarriage, prematurity, fetal growth restriction, infertility, chronic maternal diseases, lifestyle, and occupational or environmental factors.

Effective preconception care involves a range of preventive, therapeutic and behavioural interventions. Although in Italy there are national preconception care recommendations concerning the general population, they are usually encouraged informally and only for single risk factors. At present there is increasing interest in offering a global intervention in this field. The aim of this study was to investigate attitudes and behaviours of Italian women of childbearing age and healthcare professionals regarding preconception health.

**Methods:**

We conducted a qualitative study among women of childbearing age and healthcare professionals between February 2014 and February 2015. Five focus groups were held: 2 with non-pregnant women aged 22 to 44 years and 3 with healthcare professionals. Discussion topics included women’s questions about preconception health, worries and barriers regarding preconception care interventions, attitudes and behaviours of women and healthcare professionals towards preconception health, women’s information sources. In the analysis of the focus groups priority was given to what was said by the women, supplemented by information from the healthcare professionals’ focus groups.

**Results:**

Fourteen women of childbearing age (8 nulliparae and 6 multiparae) and 12 healthcare professionals (3 nurses, 4 midwives, 5 doctors) participated in the focus groups. The results indicate the presence of many barriers and a lack of awareness of preconception health relating to women, healthcare professionals and policies. Women’s knowledge and attitudes towards primary preconception care information are described. The main reference source of information in this field for Italian women seems to be their obstetric-gynaecologist.

**Conclusions:**

The study indicates that several barriers influence preconception care in Italy. Moreover, a lack of awareness of preconception health and care among Italian women of childbearing age and healthcare professionals emerges. The findings might contribute to strategies for the implementation of preconception care guidelines.

## Background

Preconception care (PC) involves health promotion to reduce risk factors that might affect the health of women and couples of childbearing age (Table 1). The risk factors of adverse reproductive outcomes (AROs) are partly known and include previous AROs, advanced maternal age, lifestyle (e.g., tobacco, alcohol use), maternal conditions (e.g., overweight and obesity), or maternal chronic diseases (e.g., pre-gestational diabetes, dysthyroidism) [1]. However, the AROs include a wide variety of conditions that negatively affect both the mother and child’s health, particularly, infertility, miscarriage, malformations, prematurity, and fetal growth restriction [1].

**Table 1:** Recommendations for preconception counselling and care

Family planning: ask women of reproductive age about intention to become pregnant. Provide contraceptive counselling tailored to patients’ intentions
Nutrition and physical activity: advise adequate fruit and vegetable intake, folic acid supplementation (400 mcg daily) to reduce the risk of neural tube defects, exercise/physical activity
Body Mass Index: assess body mass index, and counsel women who are overweight, obese, or underweight about achieving a healthy body weight before becoming pregnant
Substance use:
**-** screen for alcohol use, and provide referral for women with alcohol dependence **-** screen for tobacco use, and provide smoking cessation treatment when needed; counsel patients about the effect of smoking on pregnancies and child health **-** provide brief behavioural interventions to reduce tobacco, alcohol, and drug use
Chronic diseases: counsel women with diabetes mellitus about the importance of glycemic control before conception
Medications: assess for the use of teratogenic medications; for women with chronic diseases, switch to safer medications when possible, and use the fewest medications at the lowest dosages needed to control the disease
Communicable diseases: screen patients who wish to become pregnant for sexually transmitted infections and other communicable diseases
Immunizations: update hepatitis B, influenza, measles, mumps, rubella, tetanus, diphtheria, pertussis, varicella immunizations as needed in patients who wish to become pregnant
Family genetic history: screen for personal or family history of congenital anomalies or genetic disorders; refer couples for genetic counselling when risk factors are identified, and provide carrier testing when appropriate to determine risk to future pregnancy
Mental health: screen for depression and anxiety disorders; counsel patients about the risks of untreated depression during pregnancy, as well as the risks of treatment
Psychosocial factors: screen for intimate partner violence; evaluate the patient’s safety, and provide referral to appropriate resources
Infertility-subfertility: promote awareness and understanding of fertility and infertility and their preventable and unpreventable causes; screen couples for infertility-subfertility causes
Environmental exposures:
**-** assess for workplace exposures to toxicants; industries that are known to use toxic chemicals include clinical and laboratory health care, dry cleaning, printing, manufacturing, and agriculture **-** assess for household exposure to potentially harmful agents such as heavy metals, solvents, and pesticides **-** counsel patients about avoiding mercury exposure by not consuming large fish and limiting other fish intake
Men’s preconception counselling and care: similar to PC for women, it consists of: a reproductive life plan, nutritional and physical activity, a healthy body weight, tobacco, alcohol, drug use, exposures to teratogens, a complete medical history that includes medications, medical conditions, sexually transmitted infections screening, immunizations, family history for genetic conditions, mental health and environmental exposures

PC is a package of preventive services (screening, counselling, and management of risk factors) designed to reduce modifiable risk factors before pregnancy in order to optimize conception, pregnancy outcomes, neurodevelopmental outcomes, chronic diseases of childhood and maternal and child health. The aim of a preconception visit is to identify any risk factors for AROs in the couple that can negatively affect pregnancy and lifelong outcomes. Care in the antenatal period may be too late to address important risk factors such as obesity, tobacco or alcohol exposure.

Effective PC involves a range of preventive, therapeutic and behavioural interventions - for example, lifestyle changes, vaccinations, genetic screening, use of medicines and treatments for chronic medical conditions - to improve the pregnancy outcomes [2].

PC guidelines and recommendations have been developed in many countries [3–5] but in most European countries recommendations for healthy women and men are fragmented and inconsistent [6]. Although in Italy there are national PC recommendations concerning the general population [6], they are usually promoted informally and only for a single risk factor, such as tobacco use or specific medical conditions. However, couples often have several health and social risk factors impacting major outcomes [7].

The Euro-Peristat Project [8] indicates the need, even for Europe, to detect, address and reduce these important risk factors. In most European countries, the prevalence of risk factors for AROs is high. In particular, maternal weight before and during pregnancy affects not only the course of pregnancy and its outcome, but also the offspring’s lifelong health. More than 10% of childbearing women are obese [8] and these mothers have a higher risk of gestational diabetes and preeclampsia [9, 10]. The relative risk of stillbirths, neural tube defects or other congenital anomalies is also higher in this group and increases with the level of obesity [11–13]. In addition, macrosomia and caesarean sections are 2–3 times more common among obese women [14, 15].

Moreover, the percentage of advanced age European mothers, defined as women giving birth at 35 years or older, ranged from 10.9% in Romania to 34.7% in Italy [8]. The proportion of women bearing children later in life varies substantially. However, in 40% of countries or regions, at least 20% of births were to women aged 35 years or more, and the proportion of births in this age group increased substantially in almost every country. Late childbearing is associated with an increase in preterm birth, growth restriction, and perinatal mortality [16–18]. Older mothers have a higher risk of multiple births and a higher prevalence of pregnancy complications, including some congenital anomalies, hypertension, and diabetes. They are at a higher risk of maternal mortality and morbidity and more often give birth by caesarean section.

Furthermore, women who undergo assisted reproductive technology procedures are more likely to deliver multiple birth infants than women who conceive naturally. Multiple births pose substantial risks to both mothers and infants, including obstetric complications, preterm delivery, and low birthweight infants.

At present there is increasing interest in offering a global intervention to identify and reduce multiple risk factors, such as not taking maternal folic acid supplements before pregnancy [19], tobacco use [20], obesity [21], chronic diseases [22] and other AROs. Preconception counselling represents a valid tool to promote PC and reduce these risk factors [23–25].

The aim of this study was to investigate attitudes and behaviours of Italian women of childbearing age and healthcare professionals (HCPs) regarding preconception health in order to identify barriers and develop appropriate communication strategies to successfully promote PC.

## Methods

### Focus group discussion settings

The focus group (FG) format was chosen for this study because it is particularly useful for exploring people’s knowledge, experiences, and attitudes [26, 27].

We conducted two separate FGs with nulliparous and multiparous women. Inclusion criteria were: women aged 22–44 years, nulliparae planning a pregnancy in the following 2 years, multiparae having at least one child less than 4 years old (so they are not too far from their maternity experience). Exclusion criteria were: women with an ongoing pregnancy, women working in the healthcare field, women with previous adverse pregnancy events. Furthermore, we conducted three FGs with HCPs (nurses, midwives, obstetric-gynaecological and pediatric residents). The inclusion criterion comprised those having had work experience in a mother and child health field of at least 4 years. We decided to include multiparous women since they are more aware of unmet informational needs at the preconception stage based on their maternity experience. However, we interviewed them separately to avoid the risk of them being considered experts by nulliparous women and, thus, overshadowing their opinions. The multiparous women were asked to focus on their first preconception period. All the multiparous women but one had only one pregnancy.

Nulliparous and multiparous women were recruited through contacts with associations (3), primary care paediatricians (3), posters (2), obstetric-gynaecologists (OB-GYNs) of the Verona University Hospital (2), the assisted reproduction center (2), Facebook (1), and personal contacts (1). The HCPs were recruited through institutional channels, mainly from the Verona University Hospital.

A screening form was administered to ensure that the recruited women and HCPs were eligible to participate in the FGs according to the inclusion/exclusion criteria. Eligible and available subjects were also asked for their socio-demographic data to check the composition of the groups. The FGs took place in a meeting room within the Hospital Management Department of the Verona University Hospital. No incentives were paid to the participants.

### Focus group discussion topics

All the FGs were conducted by the same female, a trained and experienced moderator, on the basis of a semi-structured interview guide which included the following topics: women’s knowledge and questions about preconception health, attitudes and behaviours of women and HCPs towards preconception health, worries and barriers regarding PC interventions, women’s information sources (Table 2). All FGs were introduced by saying that the Center for the Control and Prevention of Diseases of the Ministry of Health had funded a project on the prevention of congenital diseases and disabilities focused on effective communication strategies. It was stated that the objective of the FG was to understand how to provide information to women about PC.

**Table 2:** List of questions for FGs and the corresponding themes

	Women^a^	HCPs
Women’s knowledge and questions about preconception health	What are the things that you are interested in knowing on the topic of motherhood: (conception and pregnancy)	Based on your experience, what is the information that women more frequently ask before pregnancy?
	What information have you received so far (and from whom)?	And instead, what should they ask and not ask?
	Is there something that your physician or other HCPs should have talked to you about and that you think they did not?	Why do you think they do not ask?
	Are there questions that you wanted to ask but for some reason did not? Why?	In your opinion, are there any questions that have not been answered? Which ones?
	What can a healthy person do to promote a good pregnancy and the health of the expected baby?	What kind of information do they seek on the Internet?
	What are the things that could affect the fetus’s development?	
	What do you know about folic acid? (When should you start taking it?)	
Attitudes and behaviours of women and HCPs towards preconception health	With whom have you spoken to regarding your intention to have a baby?	What do you think is the attitude of HCPs towards preconception interventions?
	Why? What did you expect? What did he tell you?	Why are so many women turning to the OB-GYN or services only after they get pregnant?
	What was the attitude of the GP and OB-GYN toward your questions/curiosities?	
	Why do you think so many women are turning to the OB-GYN or services only after they get pregnant or if they are having trouble getting pregnant, and not before?	
Worries and barriers regarding PC interventions	At this time what are the things that worry you most about motherhood (conception and pregnancy)?	What worries most women who intend to get pregnant?
	Have you ever thought that the child could be born with problems? This is never spoken, in your opinion, why?	
	Are there things/topics that you do not want to hear about? Why?	
Women’s information sources	Who has spoken to her GP of her intention to have a baby?	
	And to the OB-GYN?	
	What did you ask? (Or "why you did not think to talk to him/her?")	
	Have you ever sought information on the Internet on conception and pregnancy? What have you searched?	
	Are there other sources of information that you have consulted on conception and pregnancy? What kind of information have you obtained from these sources?	

^a^Please note that for multiparous women the past tense was used since the questions were referred to their preconception experience in previous pregnancy

In the last part of the FG, after all the themes in the interview guide were discussed, two one-page leaflets were distributed, each downloaded from a site dedicated to preconception health and prevention of birth defects: www.pensiamociprima.net and www.primadellagravidanza.it. Both included the following points: family planning, folic acid supplementation, mediterranean diet, body weight and physical activity, tobacco, alcohol and drugs, medications and toxic chemicals, immunizations, sexually transmitted infections, chronic diseases, and genetic disorders. The discussion of the leaflets provided additional information on knowledge, attitudes, behaviors and opinions of the participants on preconception health. Each FG lasted 1.5–2 h.

### Data management and data analysis

Each group discussion was audio-recorded and transcribed verbatim in Microsoft Word. Data were analyzed by the FG moderator from a conceptual point of view through a coding system based on the methodology proposed by Strauss and Corbin [28–30] and with the help of the Atlas.ti software (version 6.0, Atlas.ti GmbH, Berlin, Germany). In the analysis of the FGs priority was given to what was said by the women, supplemented by information from the HCP FGs.

## Results

Five FGs were carried out between February 2014 and February 2015, with Italian women of childbearing age and HCPs.

Overall 14 women of childbearing age, 8 nulliparae and 6 multiparae, participated in the FGs. The mean age for nulliparae was 36.7 years (range: 33–43); multiparae had a mean age of 34.7 years (range: 23–46) and had 1 or 2 children with an average age of 19 months. Regarding the women’s characteristics, 10 women had a university degree and 11 were married. Twelve HCPs participated in the FGs: 3 neonatal nurses, 4 hospital midwives and 5 residents (4 obstetric-gynaecological and 1 pediatric). All of them were females. The mean age of HCPs was 38.4 years (range: 29–52) with a mean work experience of 13.9 years (range: 4–32).

### Women’s knowledge and attitudes towards primary PC information

Women and HCPs agree in identifying the child’s health as the main interest and worry as soon as pregnancy is discovered: *“The child is healthy and is not affected by malformations”.* In particular Down’s syndrome is one of the main worries among the women in relation to their advanced age.

Preconception risk factors such as pre-existing conditions, overweight/obesity and infectious diseases are unknown by the participants.

Let’s summarize the knowledge and attitudes about the main topics of PC:

#### Folic acid

Six out of 8 women in the nulliparous women’s group know they have to take folic acid before pregnancy, but some of them do not understand its purpose and when or how often it should be taken. For this reason some women, after a certain period of attempts, interrupted taking it, waiting to start again once they knew they were pregnant:

*“I was also prescribed folic acid, and then I didn’t take it anymore. Maybe I’ll restart now” [nulliparous woman].*

#### Weight and diet

Many are very careful about food and aware of the risks of excessive weight gain during pregnancy; however, weight before conception was not mentioned as a concern even by overweight women.

#### Tobacco and alcohol exposure

Most of women give up smoking as soon as they are aware of pregnancy, since they know that smoking is dangerous for the baby. Alcohol instead is seen as something compatible with pregnancy:

*“Me, for example, if perhaps I will get pregnant in a year I should not drink even a glass of wine?” [nulliparous woman].*

Ambiguity in the medical prescriptions about alcohol was emphasized:

*“You have the OB-GYN who is strict and tells you "no, not even a drop of wine"; the other one who otherwise says "at your discretion." [nulliparous woman].*

#### The use of medications

Women are worried about the use of medications due to the risk of malformations. Sometimes they don’t take medications even if they have been prescribed by the physician because they are scared:

*“I’m a person who says ‘Do not take drugs!’ There’s my mom who sees me suffer but I do not want to take medications” [nulliparous woman].*

#### Genetic diseases

There is still a lot of misinformation regarding genetic testing. In some cases, women do not exclude the idea of performing genetic tests once the pregnancy is initiated, assuming that it is not possible to take preventive actions:

*“If it’s genetic, what can we do? If it is genetic there is really nothing I can do to prevent it” [nulliparous woman].*

#### Infectious diseases

In the nulliparous women’s group not all of them knew about some infectious diseases mentioned in a leaflet:

*“I don’t even know what Chlamydia is, never heard of it, and also Cytomegalovirus” [nulliparous woman].*

Very few in both women’s groups reported having had the exam for rubella or other infectious diseases before conception. However, in the HCPs’ opinions about immunizations, women just do not ask.

#### Age and chronic diseases

Risks associated with aging are not often investigated or are under-evaluated by women:

*“My desire for motherhood is not so urgent; the search has not yet started” [nulliparous woman 38 years old].*

In the experience of HCPs, women rarely stay informed about the possible consequences of their pre-existing condition on pregnancy unless they take medications for it.

### Barriers to PC

#### Lack of awareness of preconception health

Only a few women participating in the FGs went to the general practitioner (GP) or OB-GYN for PC before trying to become pregnant. The great majority turned or would have turned to the OB-GYN only after a certain period of unsuccessful attempts or after a confirmed pregnancy:

*“Actually I would have asked later, if I had gotten pregnant. I would have gone to the OB-GYN and I would have said ‘I’m pregnant, what do I have to do?’ But that moment has not arrived yet so…” [nulliparous woman].*

According to the HCPs that participated in the FGs, *“there is not a concept of preconception in Italian society”*. In the midwives’ opinions, mainly negative events (a delay in getting pregnant, a miscarriage, a history of genetic disease or cases of malformations in the family) prompt women to turn to an OB-GYN for PC. However, sometimes external advice encourages the women to have PC.

What consistently emerged in the discussions with women of childbearing age and HCPs is that women rarely ask questions before getting pregnant nor do they search for information about preconception health on the Internet. The main interest of a woman who is planning a pregnancy is *“having it or the fear of being unable to have it”*. For HCPs, as long as the women does not reach the goal, *“they have no other concerns in their minds”*.

When women become aware of their pregnancy, they ask disparate information to the OB-GYN, intensively surf the Internet, and start avoiding risky behaviours: many quit smoking, avoid eating certain foods, and begin taking precautions in the workplace in the event that they have to deal with potentially toxic substances like enamels, paints, or hair dyes.

It is also clear from the discussions that women have no idea that some problems can be prevented. The lack of knowledge is one of the main barriers to PC:

*“Since we are both healthy persons that conduct normal lives and eat a little bit of everything, I did not seek information because it didn’t seem to me that I had to do who knows what” [nulliparous women].*

*“Having a normal and healthy lifestyle, I said that possible complications of the fetus are due to genetic characteristics, not to things that I can do” [multiparous women].*

In many cases, in fact, those who do not know about the existence of certain information will not miss it, and will work with what they know. It is therefore necessary that a certain degree of information circulates for women to feel the need to ask and learn:

*“I did not look for other information on the Internet because I did not know what to look for. If I get input or receive discordant information from the OB-GYN, a friend, hearsay, then maybe I would have searched, but actually before conception I didn’t search” [multiparous woman].*

Hence women do not look for useful information on the Internet, for example, on their diets, on folic acid, on alcohol consumption, on the necessary exams, because they don’t know what to look for:

*“Indeed until you’re in a situation it’s difficult to say I’ll inform myself … I do not know what to ask and it’s a situation where maybe I’ll never be” [multiparous woman].*

Considering that HCPs might provide information on preconception health in several occasions and that the Internet represents a different source of information, we investigated the reasons why women are not aware of preconception interventions, and identified the following factors:

#### Conception as a natural event, attitude of discretion

Conception is experienced by women as a natural event of life, requiring no preparation and relating to the couple only. This is the main reason for not communicating the pregnancy planning to their OB-GYN or GPs, losing the opportunity to receive valuable information.

To the question “With whom have you spoken about your intention to have a baby?” nulliparous women answered:

*“I consider it more natural for the couple to keep this information to themselves […] It is more appropriate that they keep this information to themselves so no false expectations are given to the parents. Until there is no further need for the parents’ support because of treatments that may be underway, it might be better to keep it private”.*

*“I told no one” [nulliparous women].*

This behaviour is also a defence against possible disappointments:

*“Maybe I was also held back by fear. The desire to have children was so great that I didn’t start searching for what to do when pregnant because I would have thought about it afterwards, as a superstitious approach to this thing” [multiparous women].*

#### Lack of proactive attitude of HCPs

Frequently HCPs only provide general preconception information when directly asked by the women rather than spontaneously offer it. It emerges that, among all participants, just one woman had received preconception information from her HCP during a routine visit.

If the woman asks for information, the HCP provides it (in a more or less complete way) but if the woman does not ask for information and does not inform the HCPs that she is looking to get pregnant, very few spontaneously offer information to women of childbearing age.

In general, the impression women have is that it is not so easy to get information about preconception health and that a highly proactive attitude or a little luck is needed to get access to this information:

*“I believe that the inexperience before pregnancy is not so much related to age, in the sense that I believe at age 20, 30, 40, 50, the first time is still a bit of a leap into the unknown. There is no way, in my opinion, to come in contact with people who inform you on what may be the adequate prevention before conception, except if you have the good fortune to have friends in the healthcare field who put a bug in your ear that informs you properly and then you make physician visits specific to this” [multiparous woman].*

It seems that no one has the responsibility of offering PC. None of the HCPs involved in the study, hospital nurses, physicians and midwives, feel they are in charge of PC: they would be available in principle but they argue that they have no time due to overwhelming daily tasks.

#### Low policy priority and media carelessness

In addition to the previous factors, women and HCPs underline an insufficient interest in PC promotion by both governal health agencies and the media.

### Women’s information sources

The main reference source of information on preconception health mentioned by the women is the OB-GYN, followed by the GP. However in the obstetric-gynaecological residents’ opinion, GPs are not very informed about PC as sometimes *“they do not even know what they should mark on the prescription for the women to receive prepregnancy screening free of charge”*. Midwives and nurses do not play a role in this phase; however, in the midwives’ opinions, the communication with women would be more equal with them than with physicians.

Regarding folic acid supplements and toxoplasmosis prevention, peers and close persons, like friends, mothers and sisters, also play some role.

None of the participants searched the Internet about preconception health unless they faced problems in conceiving. However, since conception is considered something intimate, women don’t ask their physicians about this aspect until later, but rather seek information on the Internet on how to promote conception, on effectiveness and functioning of the ovulation tests, etc. The Internet is searched once they know they are pregnant. Information collected through the Internet is mainly considered for support or inspiration for further consultation with the OB-GYN, coping with simple everyday problems during pregnancy or sharing experiences. Physicians do not like when women seek information on the Internet and they feel threatened by this proactive behaviour:

*“They test you to see if you answer correctly, according to them” [physician].*

Physicians claim that women *“search the Internet until they find the answer they like, basically”*.

Table 3 describes the main results of the study.

**Table 3:** Main results of FGs with women and HCPs

Women’s knowledge and attitudes towards primary PC information	Women	HCPs
Not all the women understand the purpose and timing of folic acid supplementation	X	X
Weight before conception is not mentioned as a concern even by overweight women	X	X
Most women give up smoking as soon as they are aware of pregnancy, since they know that smoking is dangerous for the baby	X	X
A moderate intake of alcohol is seen as something compatible with pregnancy	X	X
Women are afraid to take medications during pregnancy, even when prescribed by a physician	X	X
On genetic testing, there is still a lot of misinformation	X	
Infectious diseases like Chlamydia or Cytomegalovirus are not known by nulliparae, and very few women report having had the exam for rubella or other infectious diseases before conception	X	X
Risks associated with aging are not often investigated or are underevaluated by women	X	X
Women rarely keep informed about the possible consequences of their pre-existing condition on pregnancy unless they take medications for it	X	X
Barriers to PC		
Pregnancy begins in the minds of women once they receive a positive response in a pregnancy test	X	X
There is not a concept of preconception health in the Italian population and HCPs	X	X
The main concern of a woman planning a pregnancy is fertility	X	X
Women do not know that many conditions are preventable	X	X
Women do not look for useful information because they don’t know what to look for	X	X
Conception is experienced by a woman as a natural event of life	X	X
The attitude of discretion, in some cases a defense against possible disappointments, prevents women from receiving information	X	
A lack of a proactive attitude of HCPs emerges	X	X
Women and HCPs emphasize an insufficient interest in PC promotion by governal health agencies and the media	X	X
Women’s information sources		
The main reference source of information on preconception health for Italian women seems to be the OB-GYN, followed by the GP	X	
Peers and close persons, like friends, mothers and sisters, also play some role	X	X
Before conception no information about preconception health is collected through the Internet unless there are conceiving problems	X	
Information collected through the Internet is mainly considered for support for further consultation, coping with simple everyday problems during pregnancy or sharing experiences	X	X

A substantial consistency has been found between what is reported by the women and the experience of the HCPs, although the latter tend to believe they are not responsible for the serious information gaps which emerged during the FGs.

## Discussion

The study shows the presence of many barriers to PC relating women, HCPs and policy and a lack of awareness of preconception health among Italian women and HCPs. Our findings are consistent with other studies [31–38].

To our knowledge, attitudes and reactions of Italian women of childbearing age towards the contents and approaches of PC were poorly investigated and updated information is not available. PC is also a new concept in many European countries, despite demographic and epidemiological trends showing rising levels of obesity, the development of type 2 diabetes, and delayed childbearing, all of which can lead to increased complications in pregnancy and poor outcomes [8, 9].

At present there are effective interventions before conception to reduce risk factors, such as the rubella vaccination, folic acid supplementation or PC for women with diabetes mellitus [1–6, 19–24, 39, 40]. There is less evidence of effective interventions in other areas [41].

Nonetheless, PC is not common in Italy and in Europe; therefore, it is necessary to investigate the barriers.

As already demonstrated in previous studies conducted in European countries [34, 37, 42] and the United States [43], the lack of awareness of preconception risk factors for AROs represents the main barrier. Since conception is considered to be a natural event, no need for any medical intervention or additional knowledge is required.

Regarding the awareness of preconception health, pregnancy begins in the minds of women (and sometimes even in the physicians’ minds), once they receive a positive response in a pregnancy test. Starting from that moment their attitudes change and they become very careful about their fetus’s health. The time between conception and a pregnancy diagnosis, instead, is a period of unawareness; however, once the women’s reflection on this period is stimulated with some targeted information, their interest is vivid and women admit that they don’t have the perception of the real damage one can cause during the first weeks.

Although FG participants had a medium-high level of education, information gaps about preconception health were identified. When provided with proper information, the women showed vivid interest and a need for information. Therefore, informing women about the importance of PC and embryo development immediately after conception is mandatory. In fact, explanations about these items make limitations more acceptable by women.

Regarding additional barriers, a lack of proactive attitudes by HCPs to the implementation of PC was previously investigated [44, 45] and emerged also in our FGs, together with poor efforts in promoting preconception health at a general public level. Although PC has been provided by the National Health System in Italy since 1998, very few HCPs are actively offering this service and very few women are asking for it.

Considering the pivotal role of HCPs in PC, their activity should be supported and coordinated at the local and national level and a massive information campaign might be helpful to disseminate PC awareness.

HCPs have a major role in particular in taking responsibility for the promotion of PC, at present dependent on the goodwill of HCPs. OB-GYNs and GPs probably cannot take charge of this task alone and should be supported by informational campaigns to create synergies that develop a widespread awareness on the topic. In fact the distribution of informational materials is likely to be useless if not accompanied by promotions and explanations from HCPs. A global strategy for national action was proposed against the barriers [46].

At the same time the well-developed Pregnancy Risk Assessment Monitoring System in United States [47], that collects data on maternal behaviours, experiences, health before, during and after pregnancy, and The Canadian Maternity Experiences Survey [48] indicate the importance of implementing similar systems in European countries and in Italy, to derive preventive policies [49, 50].

The results of the present study are in accordance with a previous investigation that monitored PC contents searched, published on web pages, and shared on social networks by Italian Internet users [51]. The lack of awareness of preconception health emerged in both studies. What really seems to be missing is widespread information on the fact that the risks of some birth defects can be reduced or eliminated through simple checks and interventions before pregnancy. Women don’t have the perception of the real damage that may occur in the early weeks, as well as the possibility of preventing it.

Moreover most of the participants perceived the PC as something to do when they experienced AROs and, in the midwives’ opinions, mainly negative events prompt women to turn to OB-GYNs for PC. According to initiatives that promote PC [1, 40, 52–56], it is important to consider that all women and couples can benefit from PC and, therefore, it is essential to offer the entire population a comprehensive intervention in this field.

The Internet is a source of information, especially for fertility topics, and in both contexts emerge that women in the preconception period pose questions on ways to promote conception. The main concern of a woman planning to become pregnant is just being able to conceive. Web-based monitoring system and FGs show that pregnancy begins, in fact, in the minds of women when they receive a positive response to a pregnancy test, and from that moment on, their attitude changes and they pay attention to the health of baby.

One of the limitations of the study was the homogeneity of women. The majority of our study participants were middle class, Caucasian, and with a high educational level. On the other hand it is likely that the lack of information and awareness of preconception health in women with these characteristics should be confirmed in women of other socioeconomic backgrounds, ethnicities and educational levels.

Regarding the sample size, a relatively small sample of women was recruited for this study, according to previous investigations [32, 57]. Despite conflicting suggestions for the ideal number of participants, it is argued that smaller groups can be effective for complex topics [58]. Moreover the appropriateness of the sample is assessed inductively on the basis of the achievement of the theoretical saturation [28] in examples when, during the analysis, the data become redundant and no new concepts emerge from continued sampling and data collection. From this point of view our samples proved to be adequate.

To better understand the attitudes and behaviours of women of childbearing age towards PC, both women and HCPs were involved in this study and interviewed through FGs. FGs are group interviews that feature discussions among study participants for the sake of generating data. They are a qualitative technique, focusing solely on the variety of information without any regard to quantitative aspects. The results of our study are also limited by the qualitative methodology of FGs, but the study design was considered appropriate because at this stage the goal was not to produce generalizable results, but to understand how people perceive a situation and to determine the variety of opinions.

Another potential limitation of the study was the recruitment of hospital-based nurses, midwives and physicians, but we consider that more than 70% of Italian women go to a private OB-GYN or hospital-based OB-GYN (fee-paying) to be followed during pregnancy [59]. Moreover, hospital HCPs usually perform periodic checkups on women of childbearing age.

Despite these limitations, the findings may give future direction to strategies for the implementation of PC guidelines in general practice.

## Conclusions

In conclusion, these findings indicate the presence of many barriers, a lack of awareness of preconception health by women and HCPs, and poor efforts in its promotion at a general public level. Although in Italy there are national PC recommendations regarding the general population, they are usually encouraged informally or for a single risk factor. At present it is important to offer the whole population a comprehensive intervention in this field.

These results may contribute to improve strategies for the implementation of PC guidelines in Italy. The aim is to ensure that HCPs encountering women and couples of reproductive age routinely incorporate PC in their practice.

Moreover it is essential that European countries engage in the promotion of reproductive health, making PC a priority for public health agencies and health care systems.

## Abbreviations

AROs: Adverse reproductive outcomes
FGs: Focus groups
GP: General practitioner
HCPs: Healthcare professionals
OB-GYN: Obstetric-gynaecologist
PC: Preconception care

## References

[1] Brian J, Hani KA. Preconception Health and Health Care -The clinical content of preconception care. Am J Obstet Gynecol. 2008;199: S257–396.

[2] Seshadri S, Oakeshott P, Nelson-Piercy C, Chappell LC (2012). Prepregnancy care. BMJ.

[3] Health Council of the Netherlands. Preconception care: a good beginning. Hague Health Counc Neth. The Hague, 2007; Pubblication no. 2007/19E.

[4] Royal Australian College of General Practitioners. Guidelines for preventive activities in general practice, 8^th^ edn. East Melbourne: Royal Australian College of General Practitioners, 2012.

[5] Johnson K, Posner SF, Biermann J, Cordero JF, Atrash HK, Parker CS (2006). Recommendations to improve preconception health and health care--United States. A report of the CDC/ATSDR Preconception Care Work Group and the Select Panel on Preconception Care. MMWR Recomm Rep.

[6] Shawe J, Delbaere I, Ekstrand M, Hegaard HK, Larsson M, Mastroiacovo P (2015). Preconception care policy, guidelines, recommendations and services across six European countries: Belgium (Flanders), Denmark, Italy, the Netherlands, Sweden and the United Kingdom. Eur J Contracept Reprod Health Care.

[7] Mastroiacovo P, Nilsen RM, Leoncini E, Gastaldi P, Allegri V, Boiani A (2014). Prevalence of maternal preconception risk factors: an Italian multicenter survey. Ital J Pediatr.

[8] European Perinatal Health Report: Health and Care of Pregnant Women and Babies in Europe in 2010. EURO-PERISTAT Project’s. 2013. http://www.europeristat.com/images/doc/Peristat%202013%20V2.pdf. Accessed 01 July 2015.

[9] Baci Y, Üstüner I, Keskin HL, Ersoy R, Avşar AF (2013). Effect of maternal obesity and weight gain on gestational diabetes mellitus. Gynecol Endocrinol.

[10] Ovesen P, Rasmussen S, Kesmodel U (2011). Effect of prepregnancy maternal overweight and obesity on pregnancy outcome. Obstet Gynecol.

[11] Salihu HM (2011). Maternal obesity and stillbirth. Semin Perinatol.

[12] Rasmussen SA, Chu SY, Kim SY, Schmid CH, Lau J (2008). Maternal obesity and risk of neural tube defects: a metaanalysis. Am J Obstet Gynecol.

[13] Stothard KJ, Tennant PWG, Bell R, Rankin J (2009). Maternal overweight and obesity and the risk of congenital anomalies: a systematic review and meta-analysis. JAMA.

[14] Chu SY, Kim SY, Schmid CH, Dietz PM, Callaghan WM, Lau J (2007). Maternal obesity and risk of cesarean delivery: a meta-analysis. Obes Rev.

[15] Poobalan AS, Aucott LS, Gurung T, Smith WCS, Bhattacharya S (2009). Obesity as an independent risk factor for elective and emergency caesarean delivery in nulliparous women--systematic review and meta-analysis of cohort studies. Obes Rev.

[16] Cleary-Goldman J, Malone FD, Vidaver J, Ball RH, Nyberg DA, Comstock CH (2005). Impact of maternal age on obstetric outcome. Obstet Gynecol.

[17] Huang L, Sauve R, Birkett N, Fergusson D, van Walraven C (2008). Maternal age and risk of stillbirth: a systematic review. CMAJ.

[18] Luke B, Brown MB (2007). Elevated risks of pregnancy complications and adverse outcomes with increasing maternal age. Hum Reprod.

[19] De-Regil LM, Pena-Rosas JP, Fernandez-Gaxiola AC, Rayco-Solon P. Effects and safety of periconceptional oral folate supplementation for preventing birth defects. Cochrane Database Syst Rev 2015;Issue 12.Art.No.: CD007950. DOI: 10.1002/14651858.CD007950.pub3. 10.1002/14651858.CD007950.pub3 PMC8783750 26662928

[20] Lassi ZS, Imam AM, Dean SV, Bhutta ZA (2014). Preconception care: caffeine, smoking, alcohol, drugs and other environmental chemical/radiation exposure. Reprod Health.

[21] Aune D, Saugstad OD, Henriksen T, Tonstad S (2014). Maternal Body Mass Index and the Risk of Fetal Death, Stillbirth, and Infant Death. A Systematic Review and Meta-analysis. JAMA.

[22] Wahabi HA, Alzeidan RA, Esmaeil SA (2012). Pre-pregnancy care for women with pre-gestational diabetes mellitus: a systematic review and meta-analysis. BMC Public Health.

[23] Santucci AK, Gold MA, Akers AY, Borrero S, Schwarz EB (2010). Women’s Perspectives on Counseling about Medication-Induced Birth Defects. Birth Defects Res A Clin Mol Teratol.

[24] Lassi ZS, Majeed A, Rashid S, Yakoob MY, Bhutta ZA (2013). The interconnections between maternal and newborn health - evidence and implications for policy. J Matern Fetal Neonatal Med.

[25] Dean SV, Lassi ZS, Imam AM, Bhutta ZA (2014). Preconception care: closing the gap in the continuum of care to accelerate improvements in maternal, newborn and child health. Reprod Health.

[26] Krueger RA, Casey MA. Focus groups. A Practical Guide for Applied Research. 5th edition. Thousand Oaks: SAGE Publication; 2015.

[27] Kitzinger J (1995). Qualitative research. Introducing focus groups. BMJ.

[28] Strauss AL, Corbin JM (1997). Grounded theory in practice.

[29] Miles MB, Huberman AM (1994). Qualitative Data Analysis: An Expanded Sourcebook.

[30] Patton M (1990). Qualitative evaluation and research methods.

[31] Inskip HM, Crozier SR, Godfrey KM, Borland SE, Cooper C, Robinson SM (2009). Women’s compliance with nutrition and lifestyle recommendations before pregnancy: general population cohort study. BMJ.

[32] Mazza D, Chapman A (2010). Improving the uptake of preconception care and periconceptional folate supplementation: what do women think?. BMC Public Health.

[33] Mitchell EW, Levis DM, Prue CE (2010). Preconception Health: Awareness, Planning, and Communication Among a Sample of US Men and Women. Matern Child Health J.

[34] Bitzer J, von Stenglin A, Bannemerschult R (2013). Women’s awareness and periconceptional use of folic acid: data from a large European survey. Int J Womens Health.

[35] Tuomainen H, Cross-Bardell L, Bhoday M, Qureshi N, Kai J (2013). Opportunities and challenges for enhancing preconception health in primary care: qualitative study with women from ethnically diverse communities. BMJ Open.

[36] van der Zee B, de Beaufort ID, Steegers EAP, Denktaş S (2013). Perceptions of preconception counselling among women planning a pregnancy: a qualitative study. Fam Pract.

[37] Maher M, Keriakos R (2014). Women’s Awareness of Periconceptional Use of Folic Acid Before and After Their Antenatal Visits. Clin Med Insights Womens Health.

[38] Stephenson J, Patel D, Barrett G, Howden B, Copas A, Ojukwu O (2014). How Do Women Prepare for Pregnancy? Preconception Experiences of Women Attending Antenatal Services and Views of Health Professionals. PLoS One.

[39] Bhutta ZA, Das JK, Bahl R, Lawn JE, Salam RA, Paul VK (2014). Can available interventions end preventable deaths in mothers, newborn babies, and stillbirths, and at what cost?. Lancet.

[40] World Health Organization (2013). Pre-conception care: maximizing the gains for maternal and child health. Policy brief.

[41] Whitworth M, Dowswell T (2009). Routine pre-pregnancy health promotion for improving pregnancy outcomes. Cochrane Database Syst Rev.

[42] Spence M, Alderdice FA, Harper R, McCance DR, Holmes VA (2010). An exploration of knowledge and attitudes related to pre-pregnancy care in women with diabetes. Diabet Med.

[43] Frey KA, Files JA (2006). Preconception healthcare: what women know and believe. Matern Child Health J.

[44] Mazza D, Chapman A, Michie S (2013). Barriers to the implementation of preconception care guidelines as perceived by general practitioners: a qualitative study. BMC Health Serv Res.

[45] Kachoria R, Oza-Frank R (2014). Receipt of preconception care among women with prepregnancy and gestational diabetes. Diabet Med.

[46] Mason E, Chandra-Mouli V, Baltag V, Christiansen C, Lassi ZS, Bhutta ZA (2014). Preconception care: advancing from “important to do and can be done” to “is being done and is making a difference.”. Reprod Health.

[47] Robbins CL, Zapata LB, Farr SL, Kroelinger CD, Morrow B, Ahluwalia I (2014). Core state preconception health indicators - pregnancy risk assessment monitoring system and behavioral risk factor surveillance system, 2009. MMWR Surveill Summ.

[48] Public Health Agency of Canada. What Mothers Say: The Canadian Maternity Experiences Survey. Ottawa; 2009.

[49] Connor KA, Cheng D, Strobino D, Minkovitz CS (2014). Preconception health promotion among Maryland women. Matern Child Health J.

[50] Miller EC, Liu N, Wen SW, Walker M (2011). Why do Canadian women fail to achieve optimal pre-conceptional folic acid supplementation? An observational study. J Obstet Gynaecol Can.

[51] D’Ambrosio A, Agricola E, Russo L, Gesualdo F, Pandolfi E, Bortolus R (2015). Web-based surveillance of public information needs for informing preconception interventions. PLoS ONE.

[52] Farahi N, Zolotor A (2013). Recommendations for Preconception Counseling and Care. Am Fam Physician.

[53] Centers for Disease Control and Prevention. Preconception Health and Health Care: Overview. Available at: http://www.cdc.gov/preconception/overview.html. Updated August 29, 2014. Accessed 8 Aug 2016.

[54] van Voorst S, Plasschaert S, de Jong-Potjer L, Steegers E, Denktas S (2016). Current practice of preconception care by primary caregivers in the Netherlands. Eur J Contracept Reprod Health Care.

[55] Frayne DJ, Verbiest S, Chelmow D, Clarke H, Dunlop A, Hosmer J (2016). Consensus Statement. Health Care System Measures to Advance Preconception Wellness. Consensus Recommendations of the Clinical Workgroup of the National Preconception Health and Health Care Initiative. Obstet Gynecol.

[56] Nypaver C, Arbour M, Niederegger E (2016). Preconception Care: Improving the Health of Women and Families. J Midwifery Womens Health.

[57] Adams S, Cohen E, Mahant S, Friedman JN, MacCulloch R, Nicholas DB (2013). Exploring the usefulness of comprehensive care plans for children with medical complexity (CMC): a qualitative study. BMC Pediatr.

[58] Jayasekara RS (2012). Focus groups in nursing research: methodological perspectives. Nurs Outlook.

[59] Istituto Superiore di Sanità. Percorso nascita: promozione e valutazione della qualità di modelli operativi. Le indagini del 2008–2009 e del 2010–2011. A cura di Laura Lauria, Anna Lamberti, Marta Buoncristiano, Manila Bonciani e Silvia Andreozzi 2012, Rapporti ISTISAN 12/39.

